# Fortunellin ameliorates LPS‐induced acute lung injury, inflammation, and collagen deposition by restraining the TLR4/NF‐κB/NLRP3 pathway

**DOI:** 10.1002/iid3.1164

**Published:** 2024-03-19

**Authors:** Danjuan Liu, Rongjie Guo, Bingbing Shi, Min Chen, Shuoyun Weng, Junting Weng

**Affiliations:** ^1^ Department of Critical Care Medicine the Affiliated Hospital of Putian University Putian China; ^2^ School of Ophthalmology & Optometry Wenzhou Medical University Wenzhou China

**Keywords:** ALI, apoptosis, collagen deposition, Fortunellin, inflammatory response, TLR4/NF‐κB/NLRP3

## Abstract

**Objective:**

Acute lung injury (ALI) is the prevalent respiratory disease of acute inflammation with high morbidity and mortality. Fortunellin has anti‐inflammation property, but its role in ALI remains elusive. Thus, this study clarified the function of fortunellin on ALI pathogenesis.

**Methods:**

The ALI mouse model was established by lipopolysaccharide (LPS) induction, and lung tissue damage was evaluated utilizing hematoxylin–eosin (HE) staining. The edema of lung tissue was measured by the lung wet/dry (W/D) ratio. The lung capillary permeability was reflected by the protein content in bronchoalveolar lavage fluid (BALF). Inflammatory cell infiltration was measured by the evaluation of the content of myeloperoxidase (MPO), neutrophils, and leukocytes in BALF. Cell apoptosis was measured by terminal deoxynucleotidyl transferase dUTP nick end labeling (TUNEL) assay. The secretions of inflammatory cytokines were quantified using enzyme‐linked immunosorbent assay (ELISA) assays. Lung tissue collagen deposition was evaluated by Masson staining.

**Results:**

Fortunellin attenuated LPS‐induced lung tissue damage and reduced the W/D ratio, the content of MPO in lung tissue, the total protein contents in BALF, and the neutrophils and leukocytes number. Besides, fortunellin alleviated LPS‐stimulated lung tissue apoptosis, inflammatory response, and collagen deposition. Furthermore, Fortunellin repressed the activity of the Toll‐like receptor 4 (TLR4)/nuclear factor kappa‐B (NF‐κB)/NLR Family Pyrin Domain Containing 3 (NLRP3) pathway in the LPS‐stimulated ALI model and LPS‐induced RAW264.7 cells. Moreover, fortunellin attenuated LPS‐stimulated tissue injury, apoptosis, inflammation, and collagen deposition of the lung via restraining the TLR4/NF‐κB/NLRP3 pathway.

**Conclusion:**

Fortunellin attenuated LPS‐stimulated ALI through repressing the TLR4/NF‐κB/NLRP3 pathway. Fortunellin may be a valuable drug for ALI therapy.

## Graphical abstract

Fortunellin attenuated lipopolysaccharide (LPS)‐stimulated acute lung injury (ALI) through repressing the Toll‐like receptor 4 (TLR4)/nuclear factor kappa‐B (NF‐κB)/NLR Family Pyrin Domain Containing 3 (NLRP3) pathway. Fortunellin may be a valuable drug for ALI therapy.

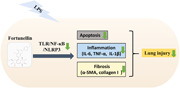

## 1 INTRODUCTION

Acute lung injury (ALI) is a severe respiratory disease with high morbidity and mortality annually.^1^ It is featured by excessive inflammation, alveolar‐capillary barrier disruption, gas exchange impairment, and pulmonary edema.^2^, ^3^, ^4^ ALI has a complex etiology and can be caused by sepsis, pneumonia, burns, chest trauma, and other factors.^5^ Despite a large number of clinical trials and research conducted, there is still no effective strategy for treating ALI due to the continuous increase in drug resistance and the emergence of new pathogens, such as the global pandemic of novel coronavirus pneumonia.^6^ Therefore, ALI remains a significant threat to public health. It is urgent to investigate new drugs for ALI therapy.

**Figure 1:**
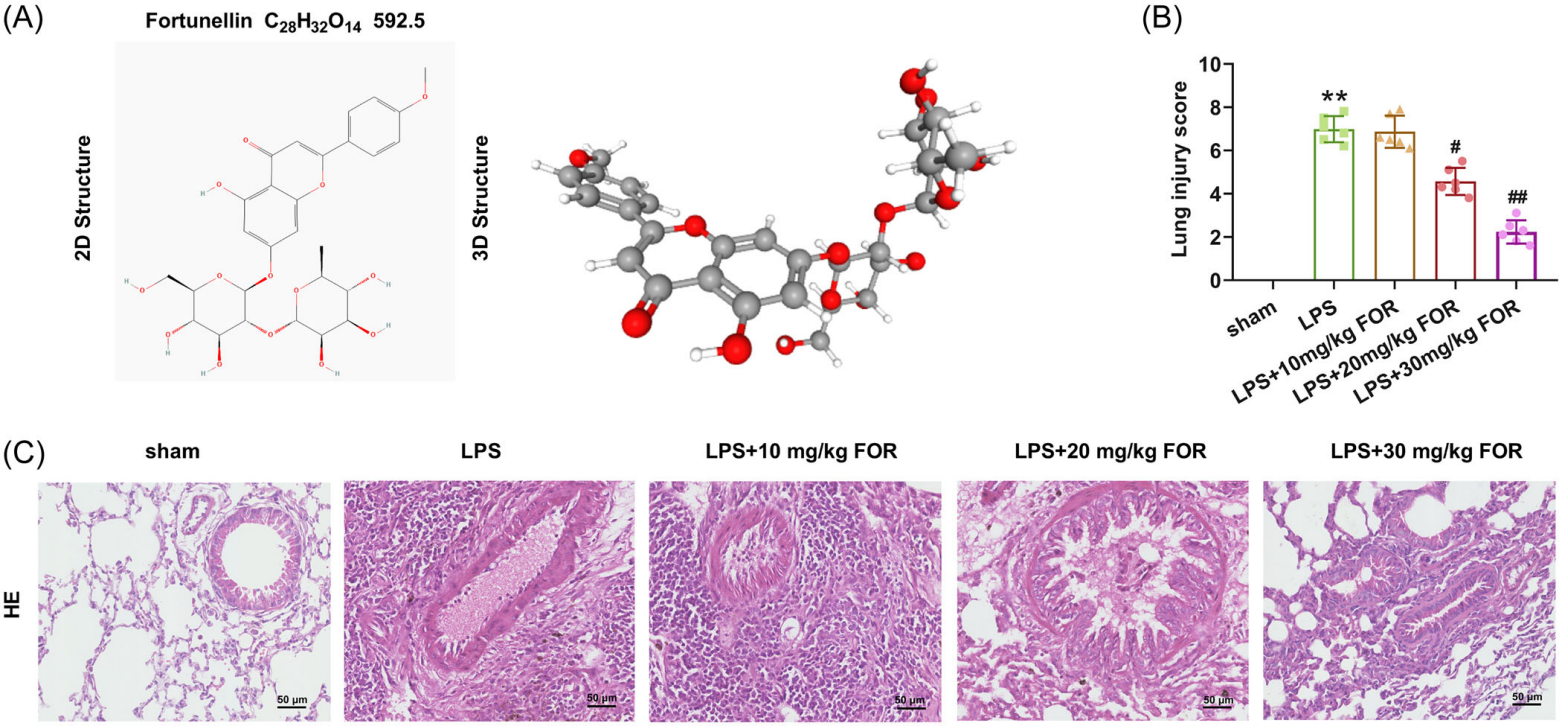
Fortunellin attenuates lipopolysaccharide (LPS)‐induced lung tissue damage. Mice were divided into sham, LPS, LPS + 10 mg/kg FOR, LPS + 20 mg/kg FOR, and LPS + 30 mg/kg FOR groups. The mice in the LPS group were treated by LPS for 12 h. The mice in LPS + FOR‐treated groups were treated with the indicated dose of fortunellin before 1 h of LPS administration. (A) The structure of fortunellin was presented. (B) The lung injury score was determined in acute lung injury (ALI) mice after treatment with 10, 20, and 30 mg/kg of fortunellin. (C) hematoxylin–eosin (HE) staining evaluated the lung tissue damage in ALI mice after treatment with 10, 20, and 30 mg/kg of fortunellin. **: *p* < .01 versus sham group; ^#^: *p* < .05 versus LPS group; ^##^: *p* < .01 versus LPS group.

Fortunellin (acacetin 7‐O‐neohesperidoside, Figure 1A) is a citrus flavonoid extracted from the fruit of *Fortunella margarita* (kumquat).^7^ Kumquat extracts usually possess anticancer, antioxidant, and anti‐inflammation properties.^8^ Accumulating evidence reveals that fortunellin has similar functions to kumquat extracts and is regarded as a valuable anti‐inflammation agent.^7^, ^9^, ^10^ For example, Zhao et al. reported that fortunellin improved the histopathological alterations and heart function and suppressed inflammatory response in diabetic cardiomyopathy.^7^ Xiong et al. found that fortunellin restrained the dysregulated inflammation and inhibited cell apoptosis in colitis.^9^ Nevertheless, the function of fortunellin on ALI remained elusive. Based on the potential function of fortunellin in inflammation regulation, we inferred that fortunellin might regulate ALI.

Toll‐like receptor 4 (TLR4)/nuclear factor kappa‐B (NF‐κB)/NLR Family Pyrin Domain Containing 3 (NLRP3) pathway is critical in the regulation of inflammation and apoptosis.^11^, ^12^ Bai et al. demonstrated that Biochanin A suppressed inflammation by restraining the TLR4/NF‐kB/NLRP3 pathway during myocardial ischemia/reperfusion injury.^11^ Luo et al. proved that Ginsenoside Rg1 ameliorated cardiomyocyte inflammation by repressing the TLR4/NF‐kB/NLRP3 pathway.^12^ Besides, this pathway also participated in the ALI progression.^13^, ^14^ For instance, glycoprotein improved lipopolysaccharide (LPS)‐stimulated ALI via blocking TLR4/NF‐κB/NLRP3 pathway.^13^ Avanafil attenuated LPS‐stimulated ALI via downregulating the TLR4/NF‐κB/NLRP3 pathway.^14^ Therefore, we speculated that fortunellin might prevent ALI progression by modulating the TLR4/NF‐κB/NLRP3 pathway.

Hence, this study aimed to elucidate whether fortunellin could alleviate ALI progression, including tissue injury, apoptosis, inflammation, and collagen deposition of the lung, in an LPS‐induced ALI model and explore if the TLR4/NF‐κB/NLRP3 pathway mediated the regulation process.

## 2 MATERIALS AND METHODS

### 2.1 Animals and treatments

C57BL/6 mice aged 6–8 weeks were acquired from Beijing Vital River Laboratory Animal Technology Co., Ltd. The animal research complied with national and international regulations and policies. This study was authorized by The Ethics Committee of the Affiliated Hospital of Putian University (No. 202013). To generate adenovirus carrying expression vectors for TLR4 (ad‐TLR4), TLR4 plasmid was blunt‐ended and cloned into the shuttle vector pAd5/CMVk‐NpA using the EcoRV site, which was completed at GenePharma.

Sixty mice were divided into five groups: sham, LPS, LPS + 10 mg/kg fortunellin (FOR), LPS + 20 mg/kg FOR, LPS + 30 mg/kg FOR groups. There are 12 mice in each group. The mice in the LPS group received intratracheal instillation of LPS (5 mg/kg) (*Escherichia coli* 055: B5, #L2880, Sigma).^15^ Briefly, mice were anesthetized with an intraperitoneal injection of 1% pentobarbital sodium, and a midline incision was made in the neck to expose the trachea. After that, a 28‐gauge needle was inserted into the trachea above the carina, and LPS was instilled. After intratracheal instillation, mice were placed vertically and rotated for 1 min to ensure even lung distribution. The mice in the sham group were given an equal volume of distilled phosphate‐buffered saline (PBS). The mice in LPS + 10 mg/kg FOR, LPS + 20 mg/kg FOR, and LPS + 30 mg/kg FOR groups received 10, 20, and 30 mg/kg of fortunellin (Purity speciation: ≥98%) (BioCrick) via intragastric administration before 1 h of LPS administration (5 mg/kg), respectively. The left lung of six mice in each group was used for bronchoalveolar lavage fluid (BALF) collection, and the right lung was used for lung wet/dry (W/D) weight ratio determination. The right lung of the other six mice in each group was used for histological analysis using Hematoxylin–eosin (HE) staining, terminal deoxynucleotidyl transferase dUTP nick end labeling staining, and Masson staining, and the left lung was used for myeloperoxidase (MPO) activity determination (left upper lobe) and western blot (left lower lobe).

The other 24 mice were allocated into sham, LPS, LPS + 30 mg/kg FOR, and LPS + 30 mg/kg FOR+ad‐TLR4 groups. There are six mice in each group. The mice in LPS + 30 mg/kg FOR+ad‐TLR4 groups were injected with ad‐TLR4 (1×10^9^ PFU/mouse) via the tail vein 7 days before other treatments. Mice were euthanasia utilizing CO_2_ inhalation at 12 h after LPS inducement. The left lung of mice in each group was used for BALF collection, and the right lung was used for histological analysis using HE staining (right upper lobe) and western blot (right lower lobe).

### 2.2 Histological analysis of lung tissue

Lung tissues were immobilized, embedded, and prepared into 5 µm slices. Then the sections were dyed utilizing hematoxylin for 5 min and eosin for 1 min. The results were evaluated using a light microscope (DM2500, Leica). The lung histopathological changes were scored using the scoring criteria as previously described.^16^

### 2.3 Lung W/D weight ratio

The right lung tissues of mice were harvested and weighed as wet weight. After that, issues were dried at 80°C for 48 h and weighed as dry weight. The wet/dry ratio was then determined to estimate the edema.

### 2.4 MPO activity determination

Lung tissues were homogenized and centrifuged at 4°C. The supernatant was harvested to evaluate the MPO activity at 460 nm utilizing the MPO Detection Kit (Nanjing Jiancheng Bioengineering Institute) in compliance with the suppliers' instructions. The results were exhibited as units per gram of total protein (U/g).

### 2.5 BALF analysis

BALF was harvested by lavaging the left lung using PBS and centrifugation. Afterward, the cell pellet and supernatant were gathered, respectively. The cell pellet was stained with Wright‐Giemesa to test neutrophils, leukocytes, and monocytes, and the numbers of neutrophils, leukocytes, and monocytes were analyzed utilizing a microscope (DM2500, Leica). The supernatant was applied to protein content analysis utilizing the bicinchoninic acid (BCA) method.

### 2.6 Lung function test

Lung function injury indexes, including lung injury score, minute ventilation (mL/kg), lung volume (mL), and airway resistance, were estimated according to the previous report.^17^

### 2.7 Terminal deoxynucleotidyl transferase dUTP nick end labeling (TUNEL) staining

The lung tissues were immobilized, embedded, and prepared into 5 µm slices. The slices were deparaffinized, rehydrated, and blocked endogenous peroxidase activity. After that, the sections were used to assess lung tissue apoptosis utilizing the Colorimetric TUNEL Apoptosis Assay Kit (Beyotime) in compliance with the supplier's protocols. The apoptosis was measured under a microscope (DM2500, Leica).

### 2.8 Western blot

Protein sample collection was performed by applying the radio‐immunoprecipitation assay lysis buffer (Beyotime), and concentration quantization was conducted using the BCA method. The harvested proteins were run on the sodium dodecyl sulfate‐polyacrylamide gel electrophoresis (SDS‐PAGE) gel and transferred to the PVDF membranes. After blocked nonspecific binding, the membranes were probed with anti‐cleaved caspase 3 (1:1000; ab231289), anti‐Bax (1:2000; ab182733), anti‐Bcl‐2 (1:2000; ab194583), anti‐α‐smooth muscle actin (α‐SMA) (1:1000; ab5694), anti‐collagen I (1:1000; ab21286), anti‐TLR4 (1:2000; SAB5700798), anti‐p‐NF‐κB (1:2000; ab264271), anti‐NF‐κB (1:2000; ab16502), anti‐NLRP3 (1:1000; ab270449), anti‐interleukin‐1β (IL‐1β) (1:2000; ab205924) and anti‐GAPDH (1:5000; ab245355) and then incubated with the Goat anti‐Rabbit IgG H&L (HRP) (1:5000; ab205718). Abcam offered all antibodies except for the anti‐TLR4 antibody (Sigma). The blots were analyzed using the electrochemiluminescence chemiluminescence system (Beyotime) and quantified using the Image J software (NIH, Bethesda).

### 2.9 Enzyme‐linked immunosorbent assay (ELISA) assays

BALF was harvested by lavaging the left lung using PBS and centrifugation at 300 g. The productions of IL‐1β, IL‐6, and tumor necrosis factor‐α (TNF‐α) in BALF of mice were detected using ELISA kits (R&D Systems) following the supplier's protocols and quantified at OD450 value immediately with the microplate reader (Multiskan FC, Thermo Fisher Scientific).

### 2.10 Masson staining

The lung tissues were immobilized, embedded, and prepared into 5 µm slices. Masson staining was then conducted on the slices to observe the deposition of collagen fibers utilizing the Trichrome Stain (Masson) Kit (Sigma) following the supplier's instruction. The staining results were analyzed under the microscope (DM2500, Leica) and quantified based on the Ashcroft score.^18^

### 2.11 Cell culture and cell transfection

RAW264.7 cells were acquired from the BeNa Culture Collection and maintained in Dulbecco's modified eagle medium with 10% fetal bovine serum (FBS) (Gibco) at 37°C with 5% CO_2_. In the indicated experiments, RAW264.7 cells were induced by 1 µg/mL of LPS for 2 h or treated by 10, 20, 40, 80, 160 μM of fortunellin for 24 h.

### 2.12 Cell viability

Cell viability of RAW264.7 cells was measured using the cholecystokinin‐octapeptide (CCK‐8) method. Cells were seeded into 96‐well plates (10^4^  cells/well) for 24 h cultivation. Then, 10 µL of CCK‐8 was added to cells for 4 h treatment. Absorbance at 450 nm was recorded utilizing the microplate reader (Thermo Fisher Scientific).

### 2.13 Cell transfection

TLR4 overexpression plasmid (pcDNA‐ROCK1) and pcDNA negative control (pcDNA‐NC) were constructed by RiboBio (RiboBio). These vectors were transfected into RAW264.7 cells utilizing Lipofectamine 3000 (Thermo Scientific) following the supplier's protocol.

### 2.14 Statistical analysis

Data were shown as mean ± standard deviation (SD), and analysis was completed using SPSS Statistics. One‐way analysis of variance (ANOVA) with Fisher's protected least significant difference post hoc test was applied for multiple comparisons. *p* < .05 was identified as statistically significant.

## 3 RESULTS

### 3.1 Fortunellin attenuates LPS‐stimulated ALI

**Figure 2:**
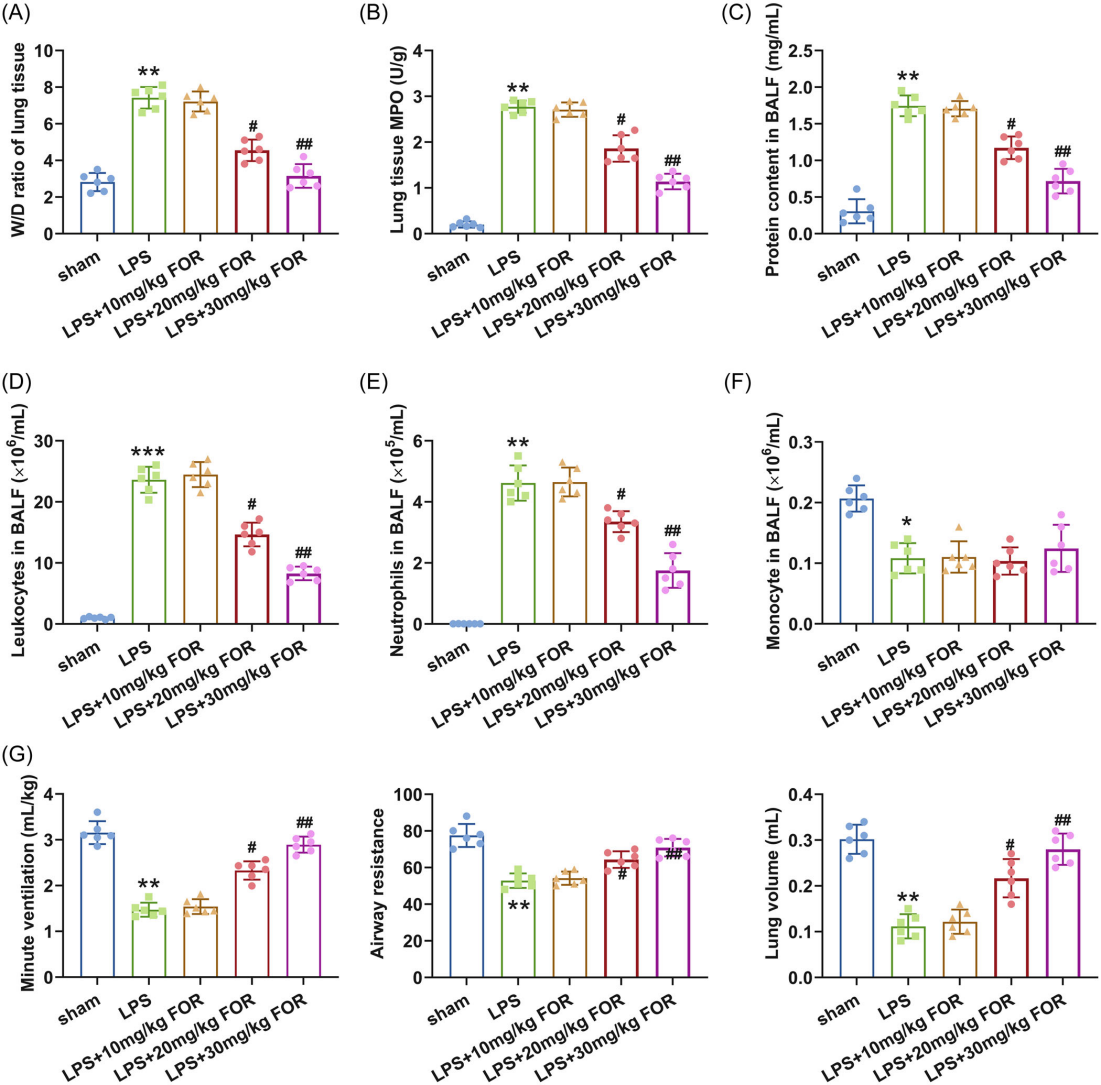
Fortunellin attenuates lipopolysaccharide (LPS)‐induced acute lung injury (ALI). The wet/dry (W/D) weight ratio of lung tissues (A), the content of myeloperoxidase (MPO) in lung tissue (B), the protein content in bronchoalveolar lavage (BALF) (C), the number of neutrophils in BALF (D), the number of leukocytes in BALF (E), the number of monocytes in BALF (F) and the minute ventilation, airway resistance, and lung volume (G) were determined in ALI mice after treated by fortunellin. **: *p* < .01 versus sham group; ***: *p* < .001 versus sham group; ^#^: *p* < .05 versus LPS group; ^##^: *p* < .01 versus LPS group.

To clarify the function of fortunellin on ALI, the ALI mouse model was first established via induction by LPS. The ALI mice model was then administrated with fortunellin. The structure of fortunellin was presented in Figure 1A. HE staining showed apparent lung tissue damage in ALI mice, while 20 and 30 mg/kg of fortunellin significantly attenuated the lung tissue damage (*p* < .05, Figure 1B,C). However, 10 mg/kg of fortunellin did not affect lung tissue damage. Besides, the W/D weight ratio of tissues and MPO activity in lung tissue were increased in ALI mice (*p* < .01), but decreased by fortunellin administration (*p* < .05, Figure 2A,B). Furthermore, the protein content in BALF and the neutrophils and leukocytes numbers were greatly unregulated in ALI mice (*p* < .01), but fortunellin treatment reversed this phenomenon (*p* < .05, Figure 2C–E). Meanwhile, the number of monocytes in BALF was slightly decreased in ALI mice (*p* < .05), while fortunellin did not affect the monocyte number (Figure 2F). Moreover, fortunellin treatment restored the decreased minute ventilation, airway resistance, and lung volume induced by LPS stimulation (*p* < .05, Figure 2G). Consequently, fortunellin attenuated LPS‐induced lung tissue impairment in mice.

### 3.2 Fortunellin attenuates LPS‐stimulated lung tissue apoptosis

**Figure 3:**
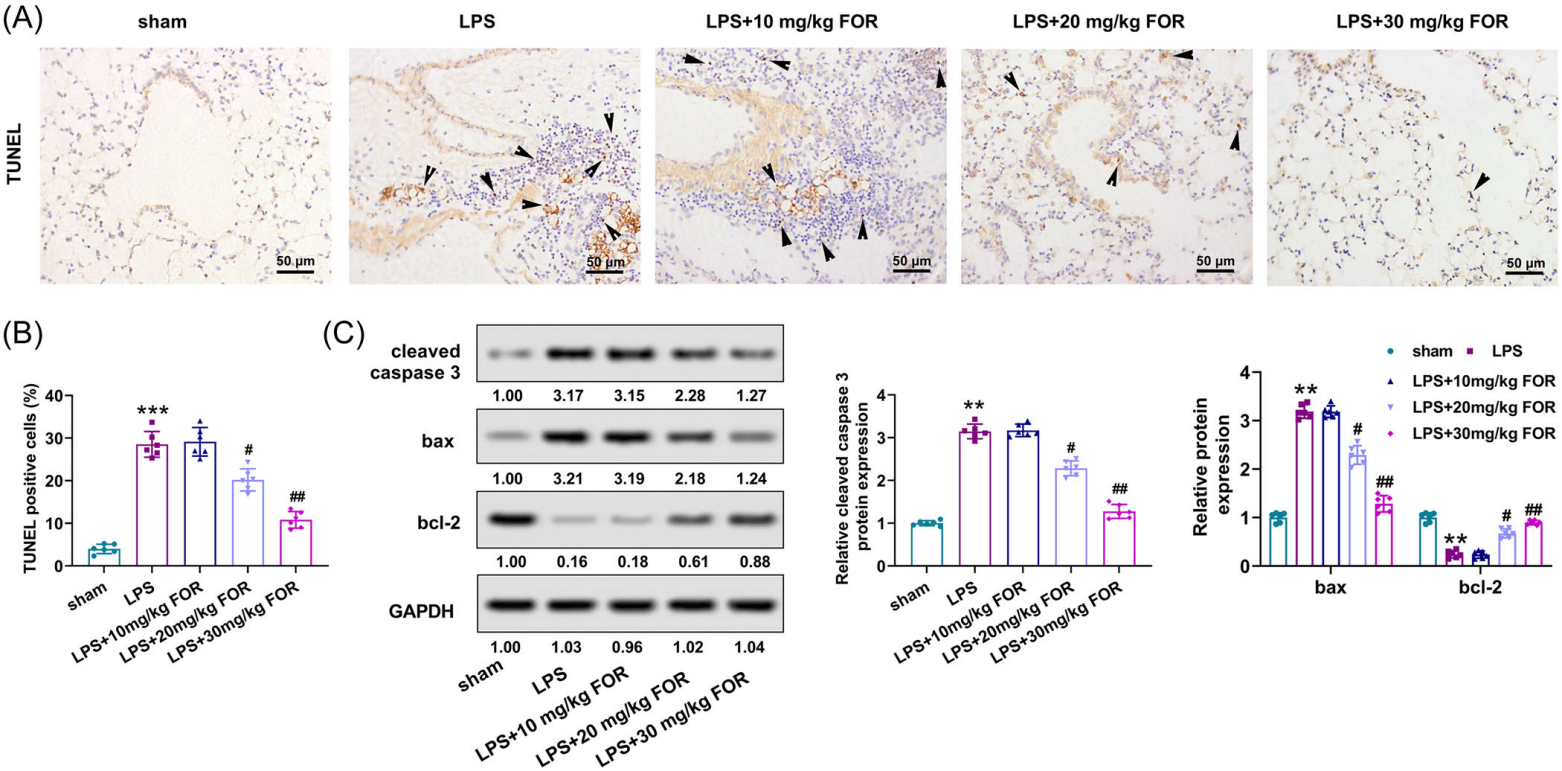
Fortunellin attenuates lipopolysaccharide (LPS)‐induced lung tissue apoptosis. (A). Cell apoptosis of lung tissues in acute lung injury (ALI) mice after being treated with fortunellin was detected using terminal deoxynucleotidyl transferase dUTP nick end labeling (TUNEL) assay. (B) The proportion of TUNEL‐positive cells was quantified. (C) The cleaved caspase‐3, Bcl‐2, and Bax levels were determined using Western blot. **: *p* < .01 versus sham group; ***: *p* < .001 versus sham group; ^#^: *p* < .05 versus LPS group; ^##^: *p* < .01 versus LPS group.

To further study the influence of fortunellin on ALI, lung tissue apoptosis in the ALI model was determined after fortunellin treatment. LPS stimulation promoted cell apoptosis in lung tissue (*p* < .001, Figure 3A,B). However, fortunellin treatment inhibited the proportion of cell apoptosis in lung tissues (*p* < .05, Figure 3A,B). Furthermore, the expressions of cleaved caspase‐3 and Bax were enhanced, but the Bcl‐2 level was downregulated after LPS stimulation (*p* < .01), which was abrogated after fortunellin administration (*p* < .05, Figure 3C). Therefore, fortunellin attenuated LPS‐induced lung tissue apoptosis in mice.

### 3.3 Fortunellin attenuates LPS‐stimulated inflammatory response and collagen deposition in lung tissue

**Figure 4:**
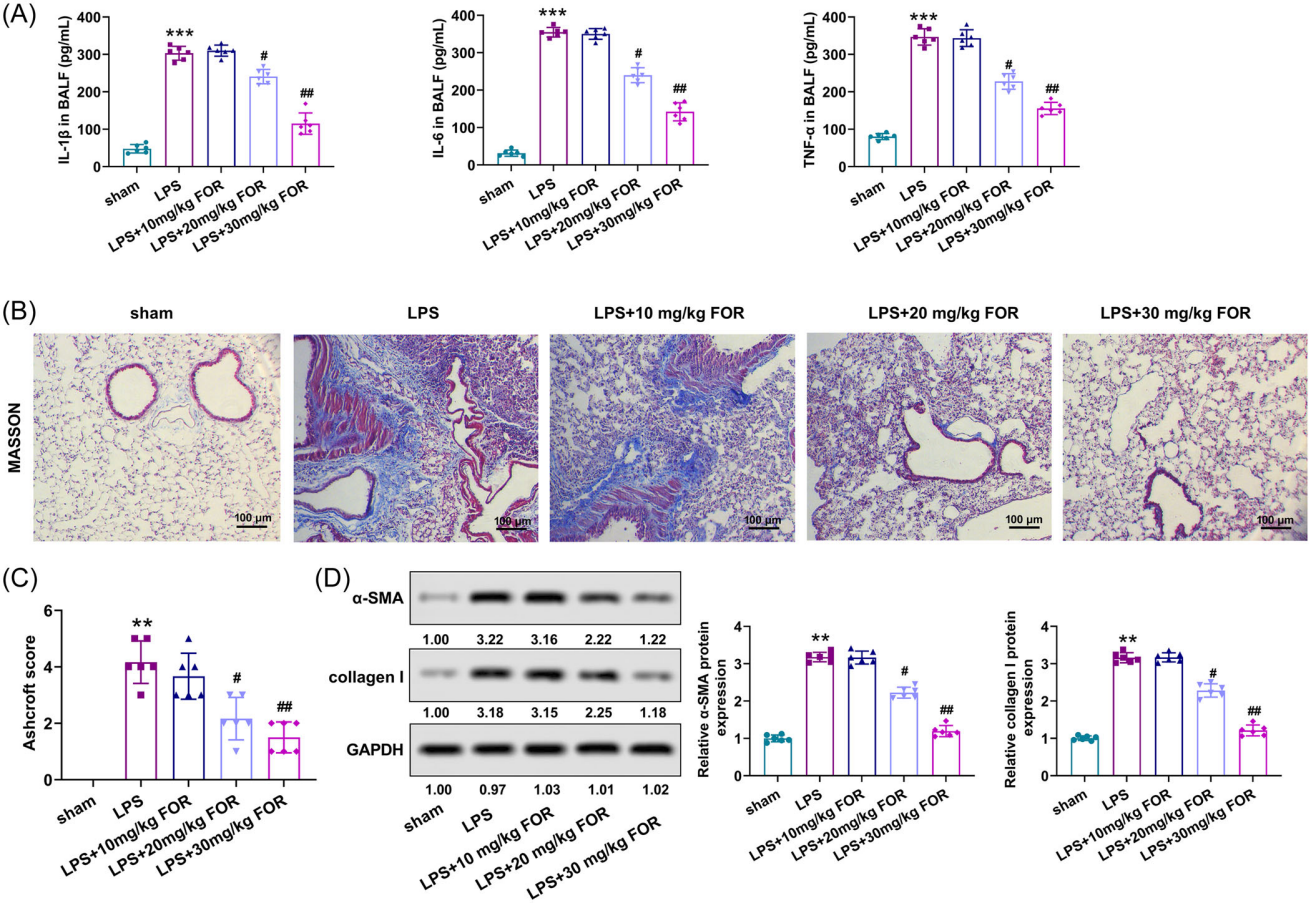
Fortunellin attenuates lipopolysaccharide (LPS)‐induced inflammatory response and fibrosis in lung tissue. (A) Interleukin‐1β (IL‐1β), IL‐6, and tumor necrosis factor‐α (TNF‐α) levels in bronchoalveolar lavage fluid (BALF) of acute lung injury (ALI) mice were detected using enzyme‐linked immunosorbent assay (ELISA) after treated with fortunellin. (B) Lung tissue fibrosis in ALI mice was evaluated using Masson staining after being treated with fortunellin. (C) Masson staining results were quantified using the Ashcroft score. (D) The levels of α‐SMA and collagen I in lung tissues of ALI mice were detected utilizing Western blot after being treated with fortunellin. **: *p* < .01 versus sham group; ***: *p* < .001 versus sham group; ^#^: *p* < .05 versus LPS group; ^##^: *p* < .01 versus LPS group.

To better elaborate on the action of fortunellin on ALI, the inflammation and collagen deposition in ALI mice were determined after fortunellin treatment. The secretions of IL‐1β, IL‐6, and TNF‐α in BALF were upregulated after LPS stimulation (*p* < .001), while fortunellin abrogated this phenomenon (*p* < .05, Figure 4A). Besides, lung tissue collagen deposition was occurred in ALI mice, and fortunellin administration attenuated the collagen deposition (Figure 4B). The Ashcroft score was consistent with Masson staining results (Figure 4C). The levels of α‐SMA and collagen I were also enhanced in lung tissues of ALI mice (*p* < .01) but reduced after fortunellin administration (*p* < .05, Figure 4D). Thus, fortunellin attenuated LPS‐induced inflammation and collagen deposition of lung tissue.

### 3.4 Fortunellin suppresses TLR4/NF‐κB/NLRP3 pathway in LPS‐stimulated ALI mice and RAW264.7 cells

**Figure 5:**
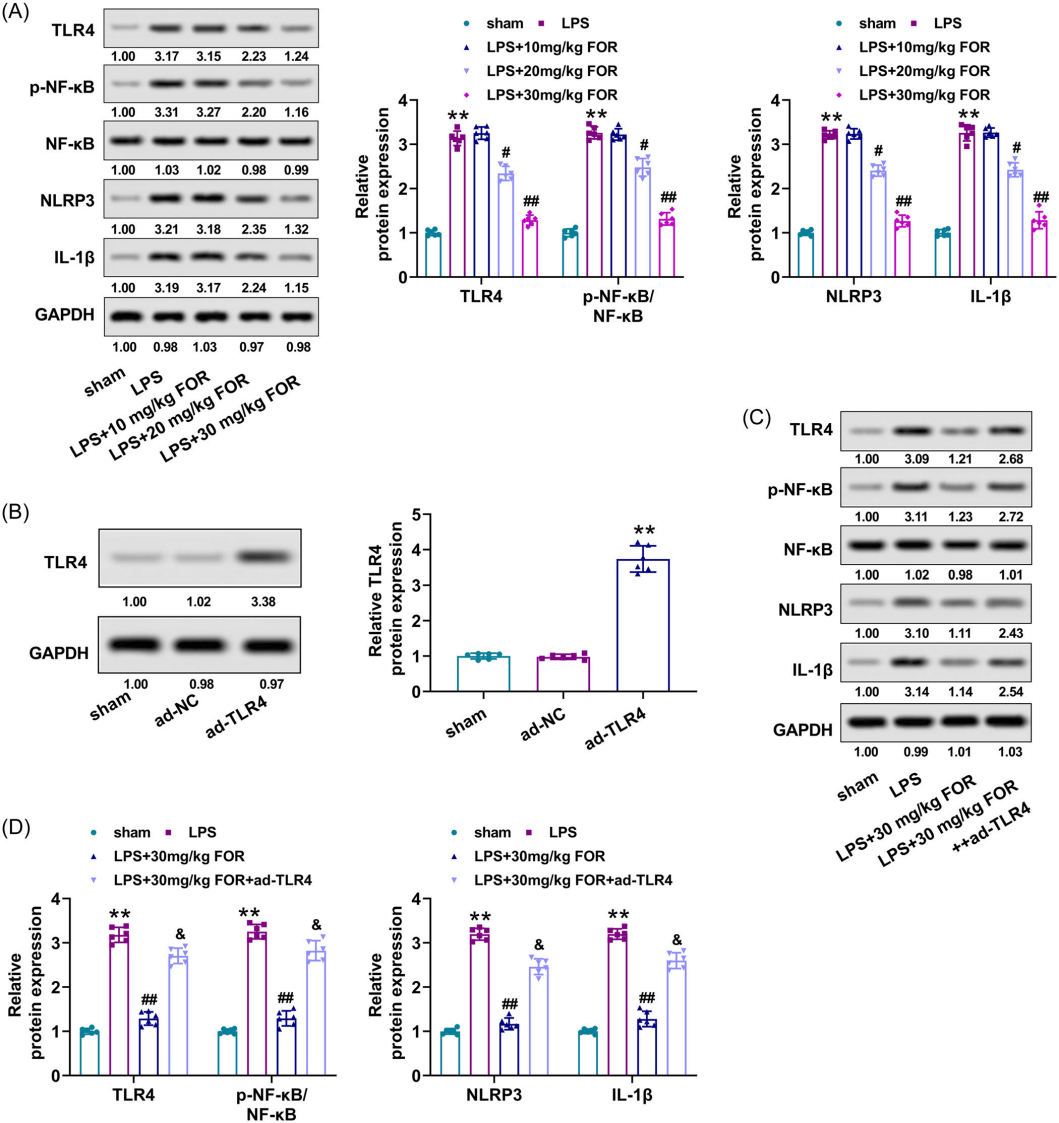
Fortunellin suppresses Toll‐like receptor 4 (TLR4)/nuclear factor kappa‐B (NF‐κB)/NLR Family Pyrin Domain Containing 3 (NLRP3) pathway in lipopolysaccharide (LPS)‐induced acute lung injury (ALI) mice. (A) The TLR4, p‐NF‐κB, NF‐κB, NLRP3, and interleukin‐1β (IL‐1β) levels in lung tissues of ALI mice were detected utilizing Western blot after treatment with fortunellin. (B) Western blot determined the level of TLR4 in lung tissues of ALI mice after injected with ad‐TLR4. Mice were divided into sham, LPS, LPS + 30 mg/kg FOR, and LPS + 30 mg/kg FOR+ad‐TLR4 groups. (C) The TLR4, p‐NF‐κB, NF‐κB, NLRP3, and IL‐1β levels in ALI mice were detected utilizing Western blot after treatment with fortunellin and overexpressed TLR4. (D) The quantification of TLR4, p‐NF‐κB, NF‐κB, NLRP3, and IL‐1β expressions. **: *p* < .01 versus sham group; ***: *p* < .001 versus sham group; ^#^: *p* < .05 versus LPS group; ^##^: *p* < .01 versus LPS group; ^&^: *p* < .05 versus LPS + 30 mg/kg fortunellin group.

To explore the mechanism of fortunellin on ALI, the TLR4/NF‐κB/NLRP3 pathway activation in ALI mice was determined after being treated with fortunellin. TLR4, p‐NF‐κB, NLRP3, and IL‐1β were elevated in lung tissues of ALI mice (*p* < .01), while fortunellin reduced the levels of these proteins (*p* < .05, Figure 5A). Afterward, the ad‐TLR4 was injected into mice to increase the TLR4 expression. ad‐TLR4 treatment greatly strengthened the TLR4 level (*p* < .01, Figure 5B). Besides, ad‐TLR4 increased the levels of fortunellin‐induced downregulation of p‐NF‐κB, NLRP3, and IL‐1β (*p* < .05, Figure 5C,D).

To further verify the action of fortunellin on the TLR4/NF‐κB/NLRP3 pathway, RAW264.7 cells were used. It was observed that 10, 20, 40, and 80 μM of fortunellin had no significant effect on the cell viability of RAW264.7 cells, while 160 μM of fortunellin decreased cell viability (Supporting Information S1: Figure 1A). Therefore, 20, 40, and 80 μM of fortunellin were selected for the subsequent experiments. Besides, LPS treatment promoted the levels of TLR4, p‐NF‐κB, NLRP3, and IL‐1β in RAW264.7 cells (*p* < .01), while fortunellin reduced the levels of these proteins (*p* < .05, Supporting Information S1: Figure 1B). Furthermore, the TLR4 overexpression vector was transfected into RAW264.7 cells to elevate the TLR4 expression, which was verified by western blot (*p* < .01, Supporting Information S1: Figure 1C). Besides, overexpressed TLR4 enhanced the levels of fortunellin‐induced downregulation of p‐NF‐κB, NLRP3, and IL‐1β in LPS‐induced RAW264.7 cells (*p* < .01, Supporting Information S1: Figure 1D). Hence, fortunellin repressed TLR4/NF‐κB/NLRP3 pathway in LPS‐stimulated ALI mice and LPS‐induced RAW264.7 cells.

### 3.5 Fortunellin attenuates LPS‐stimulated ALI via restraining the TLR4/NF‐κB/NLRP3 pathway

**Figure 6:**
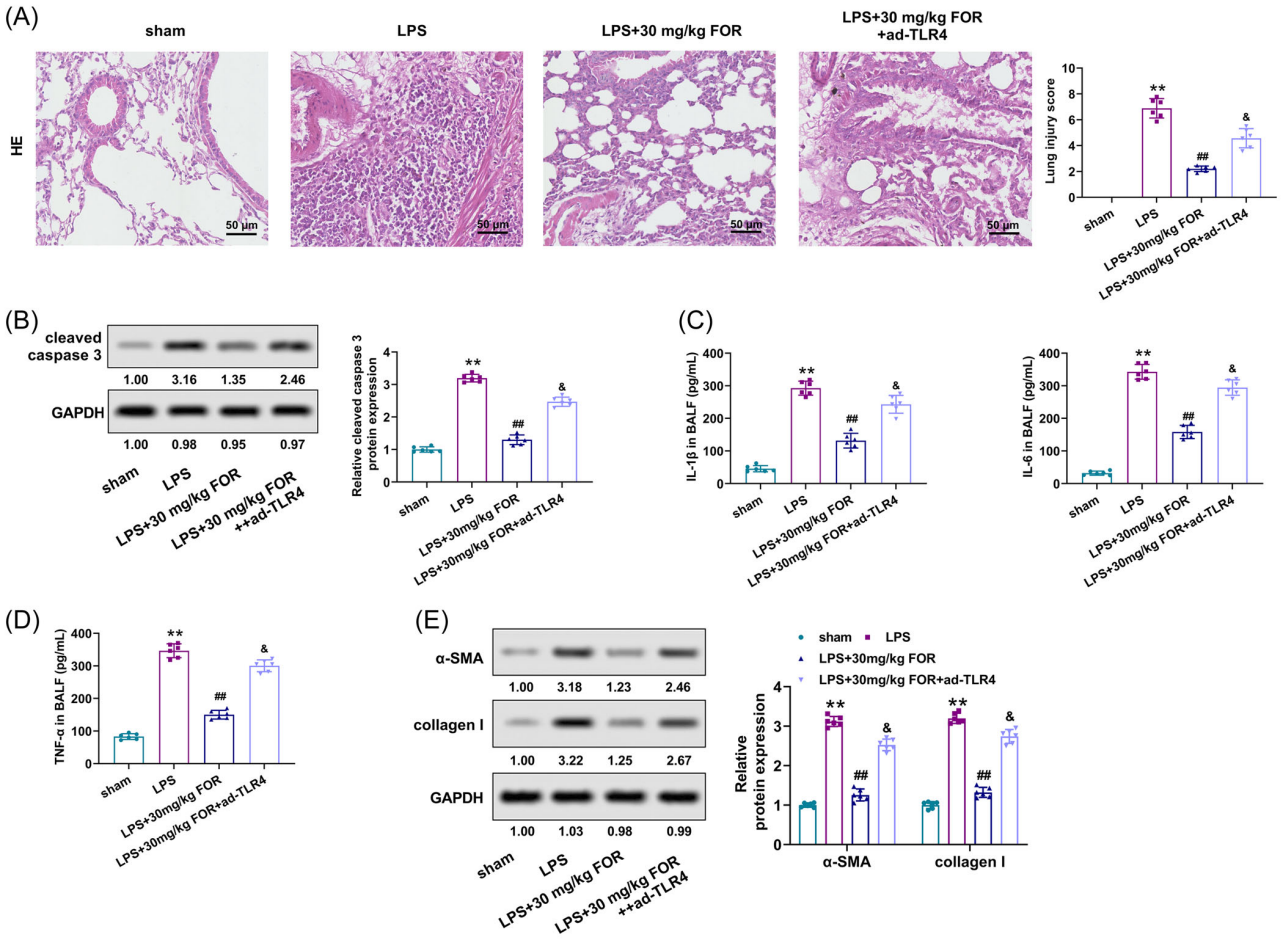
Fortunellin attenuates lipopolysaccharide (LPS)‐induced acute lung injury (ALI) by suppressing the Toll‐like receptor 4 (TLR4)/nuclear factor kappa‐B (NF‐κB)/NLR Family Pyrin Domain Containing 3 (NLRP3) pathway. (A) Hematoxylin–eosin (HE) staining evaluated the lung tissue damage in ALI mice after treated with fortunellin and overexpressed TLR4. (B) The level of cleaved caspase‐3 in lung tissues of ALI mice was determined using Western blot after treated with fortunellin and overexpressed TLR4. (C,D) Interleukin‐1β (IL‐1β), IL‐6, and tumor necrosis factor‐α (TNF‐α) levels in bronchoalveolar lavage fluid (BALF) of ALI mice were detected using enzyme‐linked immunosorbent assay (ELISA) after treated with fortunellin and overexpressed TLR4. (E) The levels of α‐SMA and collagen I in lung tissues of ALI mice were detected utilizing Western blot after being treated with fortunellin and overexpressed TLR4. **: *p* < .01 versus sham group; ^##^: *p* < .01 versus LPS group; ^&^: *p* < .05 versus LPS + 30 mg/kg fortunellin group.

To confirm whether fortunellin regulated ALI through modulating the TLR4/NF‐κB/NLRP3 pathway, the lung tissue changes of ALI mice were determined after being treated by fortunellin and overexpressed TLR4. Results found that the protection of fortunellin on lung tissue damage in ALI mice was abolished by overexpressed TLR4 (*p* < .05, Figure 6A). Besides, the inhibitory influences of fortunellin on the cleaved caspase‐3 in lung tissue and the secretions of IL‐β, IL‐6, and TNF‐α in BALF of ALI mice were also reversed by overexpressed TLR4 (*p* < .05, Figure 6B–D). Furthermore, overexpressed TLR4 abrogated the alleviative function of fortunellin on the levels of α‐SMA and collagen I in lung tissues of the ALI model (*p* < .05, Figure 6E). Collectively, fortunellin attenuated LPS‐stimulated ALI by blocking the TLR4/NF‐κB/NLRP3 pathway.

## 4 DISCUSSION

ALI is the prevalent respiratory disease of acute inflammation with high morbidity and mortality.^1^, ^2^ An effective strategy for ALI therapy still needs to be developed due to the continuously increasing drug resistance and the emergence of new pathogens.^6^ Fortunellin has anti‐inflammation properties,^7^, ^9^, ^10^ while its role in ALI progression remains elusive. Hence, we investigated the function of fortunellin on ALI development and studied the potential mechanism.

LPS is the principal part of the outer membranes of gram‐negative bacteria, and it triggers lung impairment and inflammation.^19^ The LPS‐stimulated ALI model is accepted for studying ALI because it simulates the pathological events in ALI progression, including inflammation and histological changes.^19^, ^20^ Therefore, the LPS‐induced ALI model was selected to perform follow‐up experiments in this study. In this model, the lung tissue structure was severely damaged, reflected by destroyed pulmonary architecture, inflammatory cell infiltration, and intra‐alveolar edema, consistent with the previous study.^19^ Besides, the W/D weight ratio of lung tissues, MPO activity in lung tissue, the protein content in BALF, and the neutrophils and leukocytes numbers in BALF were increased in ALI mice. The W/D weight ratio can assess the magnitude of edema.^6^ MPO content reflects neutrophil infiltration.^19^ These results confirmed lung tissue damage, edema, and inflammatory cell infiltration in ALI mice. These findings indicated that the LPS‐stimulated ALI mice model was effectively constructed. Furthermore, we found that the LPS‐stimulated lung impairment, edema, and inflammatory cell infiltration were alleviated by fortunellin administration.

Apoptosis, a form of programmed cell death, is imperative for the selective clearance of cells.^21^ Pulmonary cell apoptosis exerts a vital role in ALI pathogenesis.^21^ In many studies, cell apoptosis occurred during ALI.^22^, ^23^, ^24^ Chen et al. found that cell apoptosis was enhanced in the lung tissues of ALI mice.^22^ Similarly, cell apoptosis of lung tissues was observed in this study. Interestingly, the promoted cell apoptosis triggered by LPS was suppressed after fortunellin treatment. This finding was consistent with the reported studies.^9^, ^10^ Xiong et al. revealed that fortunellin repressed epithelial cell apoptosis by controlling phosphatase and tensin homolog deleted on chromosome 10 expression in colitis.^9^ Agrawal et al. predicted that fortunellin might inhibit the apoptosis pathway and protect against tissue damage in severe acute respiratory syndrome coronavirus 2 (SARS‐CoV‐2) infected tissues.^10^ Thus, these findings revealed that fortunellin attenuated LPS‐induced lung tissue apoptosis.

The excessive inflammatory response is a primary feature of ALI.^25^ In LPS‐stimulated ALI, TLR4 identifies LPS and subsequently facilitates the NF‐κB activation.^21^ NF‐κB activation triggers the secretions of inflammatory cytokines, such as TNF‐α, IL‐6, and IL‐1β.^21^ In the present research, the elevated secretions of TNF‐α, IL‐6, and IL‐1β in ALI mice were reduced by fortunellin. In other words, fortunellin suppressed the inflammation stimulated by LPS. Similarly, Zhao et al. found that the proinflammatory cytokines were dramatically reduced by fortunellin in high fructose‐induced diabetic heart injury.^7^ Xiong et al. demonstrated that fortunellin inhibited excessive inflammation in colitis.^9^ Collagen deposition was the feature of fibroproliferation which is a part of the normal repair response process, and patients may develop fibrosis if this process is ineffective or persists unabated.^26^, ^27^ Recent evidence revealed that excessive lung collagen deposition in lung tissue usually occurred after ALI, even at the onset of ALI, and contributed to lung fibrosis.^28^, ^29^ In this study, lung tissue collagen deposition was also observed, which is consistent with previous studies.^30^, ^31^ Besides, fortunellin reduced lung tissue collagen deposition. Together, these findings revealed that fortunellin attenuated LPS‐induced inflammatory response and collagen deposition of lung tissue.

**Figure 7:**
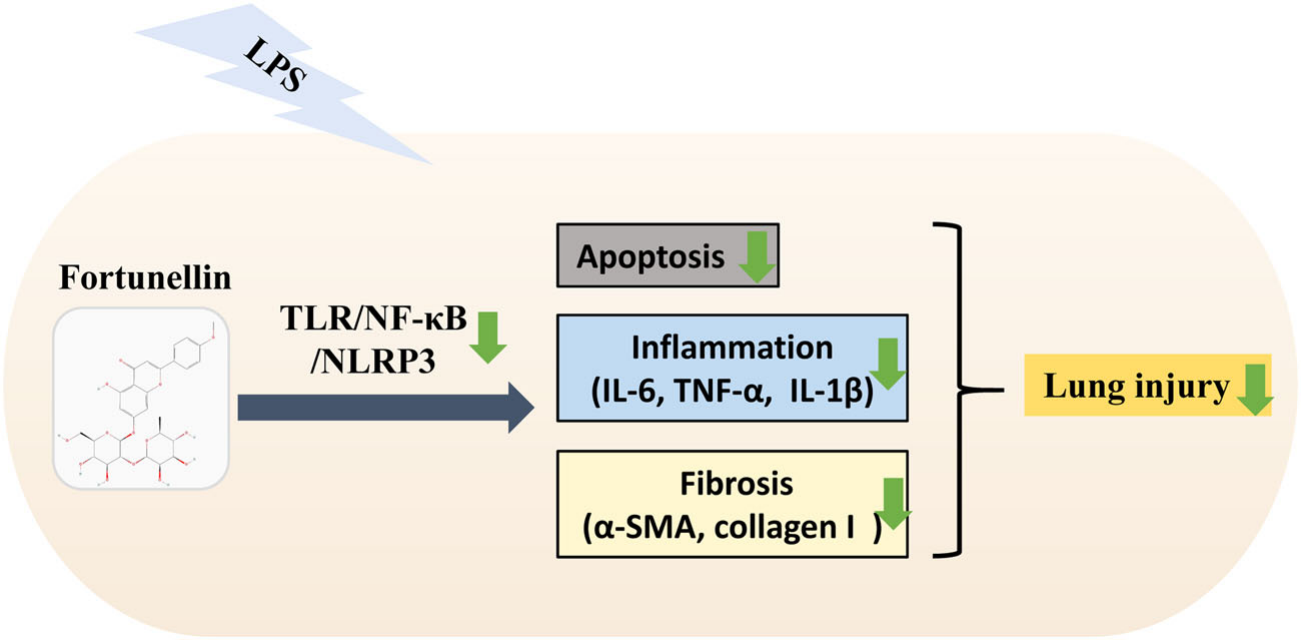
Proposed mechanism of fortunellin on lipopolysaccharide (LPS)‐stimulated acute lung injury (ALI). Fortunellin attenuates LPS‐induced apoptosis, inflammation, and fibrosis through restraining the Toll‐like receptor 4 (TLR4)/nuclear factor kappa‐B (NF‐κB)/NLR Family Pyrin Domain Containing 3 (NLRP3) pathway.

TLR4/NF‐κB/NLRP3 pathway is vital in the regulation of inflammation and apoptosis processes.^11^, ^12^ TLR4 can recognize the LPS and activate NF‐κB, which modulates the secretions of inflammatory cytokines.^21^ Furthermore, NLRP3 accumulation in the lung during LPS exaggerates the inflammation reaction.^32^ The published studies proved that inhibition of TLR4/NF‐κB/NLRP3 pathway activation attenuated ALI injury.^13^, ^14^ Niu et al. reported that glycoprotein mitigated LPS‐stimulated ALI via blocking TLR4/NF‐κB/NLRP3 signaling pathway.^13^ As expected, our research clarified that fortunellin restrained the TLR4/NF‐κB/NLRP3 pathway activation in LPS‐stimulated ALI and LPS‐induced RAW264.7 cells. Furthermore, the gain‐of‐function assay revealed that fortunellin attenuated LPS‐stimulated lung impairment, apoptosis, inflammation, and collagen deposition via restraining the TLR4/NF‐κB/NLRP3 pathway. In brief, fortunellin protects against LPS‐stimulated ALI by blocking the TLR4/NF‐κB/NLRP3 pathway (Figure 7).

In conclusion, fortunellin attenuates LPS‐induced lung impairment, apoptosis, inflammation, and collagen deposition by restraining the TLR4/NF‐κB/NLRP3 pathway. This research reported the function of fortunellin on LPS‐stimulated ALI pathogenesis and elaborated on the mechanism. Fortunellin may be a valuable drug for ALI therapy. The limitation of this study is that we did not investigate the effect of fortunellin on oxidative stress and autophagy in ALI, which will be explored in the following study.

## AUTHOR CONTRIBUTIONS

Junting Weng, Bingbing Shi, Min Chen, Shuoyun Weng, and Danjuan Liu conceived and designed the study. Junting Weng, Bingbing Shi, Shuoyun Weng, and Danjuan Liu performed the experiments and collected the data. Junting Weng, Rongjie Guo, Bingbing Shi, and Danjuan Liu performed statistical analysis. Junting Weng, Rongjie Guo, Bingbing Shi, Min Chen, and Danjuan Liu wrote the manuscript.

## CONFLICT OF INTEREST STATEMENT

The authors declare no conflict of interest.

## ETHICS STATEMENT

This study was authorized by The Ethics Committee of the Affiliated Hospital of Putian University (No. 202013).

## Supporting information


**Supplementary Figure 1. Fortunellin suppresses TLR4/NF‐κB/NLRP3 pathway in LPS‐induced RAW264.7 cells. A.** Cell viability of RAW264.7 cells after treated with different doses of fortunellin was determined using the CCK‐8 method. **B.** The levels of TLR4, p‐NF‐κB, NF‐κB, NLRP3, and IL‐1β in LPS‐induced RAW264.7 cells were detected utilizing Western blot after treatment with fortunellin. **C.** Western blot determined the level of TLR4 in RAW264.7 cells after transfected with the TLR4 overexpression vector. **D.** TLR4, p‐NF‐κB, NF‐κB, NLRP3, and IL‐1β levels in LPS‐induced RAW264.7 cells were detected utilizing Western blot after treatment with fortunellin and overexpressed TLR4. **: *P* < 0.01 versus control group; ^#^: *P* < 0.05 versus LPS group; ^##^: *P* < 0.01 versus LPS group; ^&^: *P* < 0.05 versus LPS + 80 μM fortunellin group.

## Data Availability

The figures used to support the findings of this study are included in the article.
