# Supplementary figures and images for: Fortunellin ameliorates LPS‐induced acute lung injury, inflammation, and collagen deposition by restraining the TLR4/NF‐κB/NLRP3 pathway
Source: Immun Inflamm Dis. 2024 Mar 19;12(3):e1164. doi: 10.1002/iid3.1164 (PMC10949398; doi:10.1002/iid3.1164)

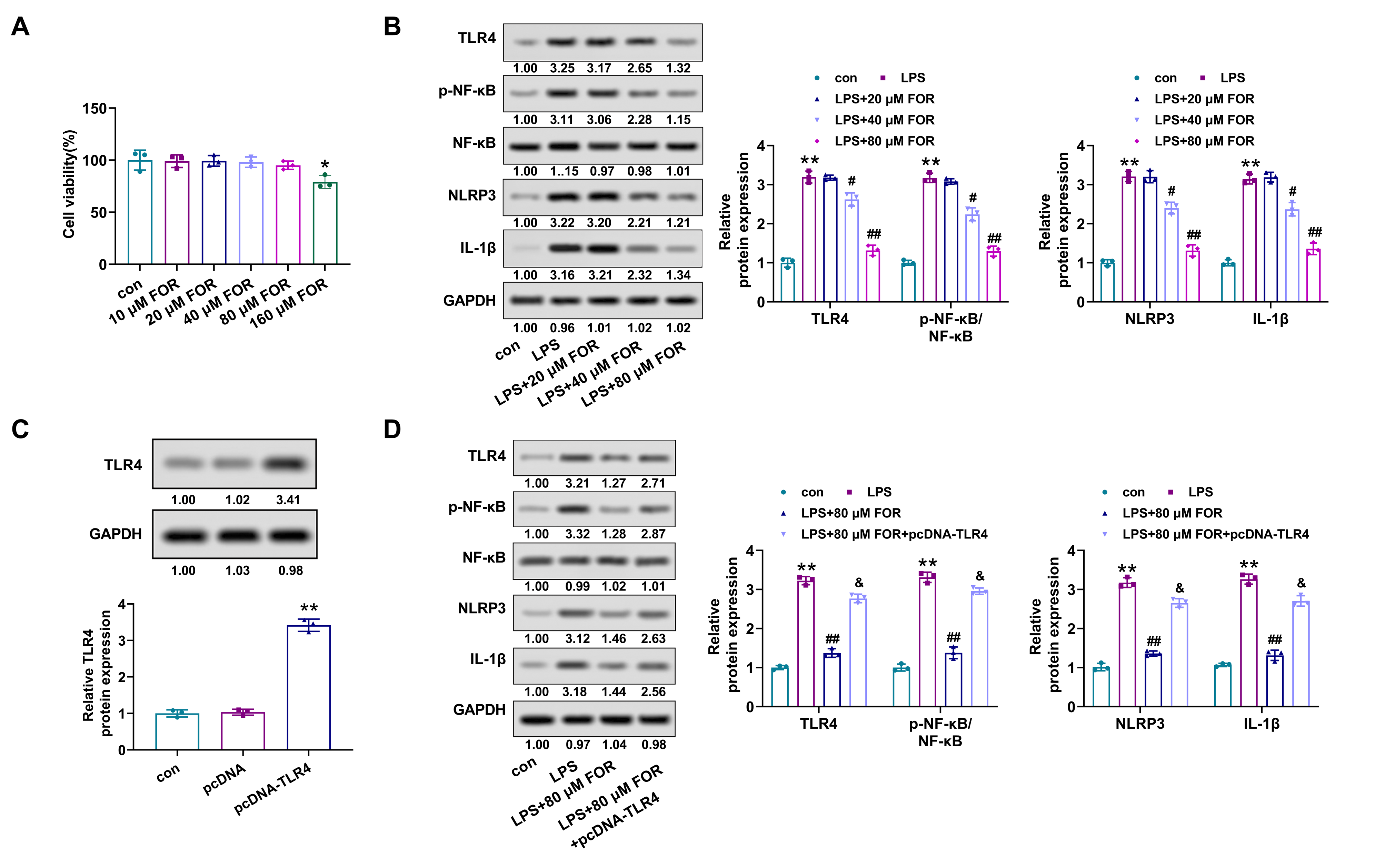

Supplement: Supplementary file 1 — Supplementary Figure 1. Fortunellin suppresses TLR4/NF‐κB/NLRP3 pathway in LPS‐induced RAW264.7 cells. A. Cell viability of RAW264.7 cells after treated with different doses of fortunellin was determined using the CCK‐8 method. B. The levels of TLR4, p‐NF‐κB, NF‐κB, NLRP3, and IL‐1β in LPS‐induced RAW264.7 cells were detected utilizing Western blot after treatment with fortunellin. C. Western blot determined the level of TLR4 in RAW264.7 cells after transfected with the TLR4 overexpression vector. D. TLR4, p‐NF‐κB, NF‐κB, NLRP3, and IL‐1β levels in LPS‐induced RAW264.7 cells were detected utilizing Western blot after treatment with fortunellin and overexpressed TLR4. **: P < 0.01 versus control group; #: P < 0.05 versus LPS group; ##: P < 0.01 versus LPS group; &: P < 0.05 versus LPS + 80 μM fortunellin group. [file IID3-12-e1164-s001.tif]
